# Assessing Hand Hygiene and Low-Level Disinfection of Equipment Compliance in an Acute Care Setting: Mixed Methods Approach

**DOI:** 10.2196/18788

**Published:** 2020-06-05

**Authors:** Hammad Akram, Alison Andrews-Paul, Rachel Washburn

**Affiliations:** 1 Baylor Scott and White Medical Center-Hillcrest Waco, TX United States; 2 Department of Public Health Baylor University Waco, TX United States

**Keywords:** compliance, hand hygiene, low-level disinfection, patient safety, qualitative, quality improvement, infection prevention, infection control, nursing, mixed-methods, HAI, health care-associated infections, hand washing, hand sanitizers

## Abstract

**Background:**

Hand hygiene and low-level disinfection of equipment behaviors among hospital staff are some of the leading cost-effective methods to reduce hospital-acquired infections (HAI) among patients.

**Objective:**

The aim of this study is to examine hand hygiene and low-level disinfection of equipment practices in a central Texas hospital and to explore pertaining gaps, perceptions, and challenges.

**Methods:**

Data were collected using a multipronged mixed methods approach that included the following: (1) observation of hand hygiene and low-level disinfection practices (12 and 8 units during morning and evening shifts, respectively); (2) observation of usability/placement of hand sanitizer dispensers; (3) semistructured interviews; and (4) a follow-up email survey.

**Results:**

In total, 222 (156 morning shift and 66 evening shift) staff members were observed. Of 526 hand hygiene and 33 low-level disinfection opportunities, compliance was observed 410 (78%) and 17 (51%) times, respectively. Overall, 6 units (50%) had ≥0.80 (favorable) hand hygiene compliance during the morning shift and 2 units (25%) had ≥0.80 hand hygiene compliance during the evening shift. Aggregated low-level disinfection compliance was 0.54 during the morning and 0.33 during the evening. Overall, the odds of noncompliant hand hygiene behavior were 1.4 times higher among staff who worked during night shifts compared to day shifts; however, this relationship was not statistically significant (95% CI 0.86-2.18; *P*=.18). Noncompliant behavior was most likely among unit B staff during the evening; however, this relationship was not statistically significant (OR 5.3, 95% CI 0.84-32.9; *P*=.07) All units, except one, had similar hand sanitizer dispenser usability characteristics. In the qualitative part of the study, the following challenges were identified: “shortage of time while seeing patients,” “sometimes the staff forgets,” “concern about drying hands,” “behavior is difficult or requires reminders,” and “there may be issues with resources or access to supplies to perform these behaviors.” Staff also stated that “a process that is considered effective is the Stop the Line program,” and that the “behavior is easy and automatic.”

**Conclusions:**

Hand hygiene and low-level disinfection compliance is dependent on several personal and nonpersonal factors. Issues such as time constraints, peer pressure, work culture, available resources, and understanding of guidelines could influence staff behavior. The information collected through this study can be used to re-examine similar or related issues at a larger scale.

## Introduction

Hand hygiene and low-level disinfection of equipment behaviors among hospital staff are some of the leading cost-effective methods in reducing hospital-acquired infections (HAI) among patients [1-3]. Low compliance with hand hygiene practices and protocols among health professionals is common and, in some instances, found to be less than 50% [1,2]. A North Carolinian study showed a 6% reduction in the rate of overall HAI, a 14% reduction in the rate of hospital-acquired *Clostridioides difficile* infections (CDI), and significant cost reductions due to high hand hygiene compliance [4]. This is a strong indicator that maintaining a high level of compliance to infection prevention protocols should be paramount to hospital processes. Nosocomial infections are one of the most preventable types of disease. It is widely accepted that maintaining hand hygiene among health professionals is a highly effective way to reduce the transmission of virulent bacteria [5].

Baylor Scott & White Medical Center-Hillcrest (also known as Hillcrest Hospital) is a 236-bed acute care facility in Waco, Texas. Rates of HAI that could be prevented by hand hygiene and low-level disinfection of equipment practices at Hillcrest Hospital are comparable to the national average [6]. From April to December 2017, Hillcrest’s Standard Infection Ratio (SIR), which expresses the reported number of infections compared to the predicted number of infections, was 0.945 for MRSA and 0.885 for CDI [6]. There were 4 reportable cases of MRSA and 27 reportable cases of CDI during this same time period [6]. This motivated us to examine hand hygiene and low-level disinfection of equipment behaviors in a health care facility to establish baseline information pertaining to these practices.

Hand hygiene guidelines include decontaminating hands before and after entering a patient’s room by using a handwashing technique or, if hands are not visibly soiled, an alcohol-based hand sanitizer [7]. Hand hygiene techniques should also be performed before donning gloves, before direct contact with patients, after contact with inanimate objects, and after contact with any body fluids or excretions [7]. Environmental and equipment cleaning guidelines include the cleaning and disinfection of patient care equipment, portable patient care equipment, and computers on wheels. Noncritical nonelectric equipment such as wheelchairs and crutches should be cleaned and disinfected after each patient use and whenever visibly soiled. Noncritical electric equipment, such as blood pressure and oximetry monitors, should be cleaned and disinfected after each patient use and whenever visibly soiled [7]. These guidelines are mandated by the Infection Prevention and Control staff at Hillcrest Hospital.

This article highlights the situation of hand hygiene and low-level disinfection practices at Hillcrest. The aim of this quality improvement project was to identify and understand associated gaps, staff perceptions, challenges, and resource-related issues that could affect and improve health care and nursing staff’s performance and compliance.

## Methods

### Hand Hygiene and Low-Level Disinfection Compliance Data Collection

The data collection strategy was modeled on a method that was previously used by Cure and Van Enk [8]. The hand hygiene and low-level disinfection observations took place at 12 units during the morning shift and 8 units during the evening shift. The study was conducted in inpatient medical and/or surgery, surgical and medical critical, emergency, women and children, and rehabilitation units from May to June 2019 by an observer that spent 1 hour at each unit. The “Observations” indicate the number of times the investigator observed compliant behavior (using hand sanitizer, washing hands, or cleaning equipment) and “Opportunities” correspond to the number of opportunities or instances in which compliant behavior should have been practiced. The compliance rate was then calculated by using the following equation:

Compliance rate (CR)=Number of times staff followed appropriate behavior / Total number of observed opportunities

If the door to a room was closed or the staff member was out of view, the behavior was not documented.

### Hand Sanitizer Dispenser Placement (Usability) Factors

The usability of hand sanitizer dispensers was measured based on the criteria described elsewhere [8] and comprised the following: (1) easily visible on entry, (2) easy, unobstructed access, (3) close to the point of care, (4) visible from point of care, (5) along the workflow path, (6) close to the entrance or exit, and (7) placed at optimal height (85 to 110 centimeters). A final criterion, (8) visible on exit, was also added. 

### Semistructured Interviews

Semistructured interviews were modeled using the existing Theoretical Domains Framework [9,10]. This framework was created to help assess the potential factors that may influence the behavior of health care professionals. The interview questions (Multimedia Appendix 1) were derived from a succinct table describing the 12 domain details, which are available elsewhere [9]. In total, 4 staff members were consented and interviewed, 2 each from units with ≥0.80 and ≤0.80 compliance, respectively. The Theoretical Domains Framework is useful for studying the implementation of desired behaviors among health care professionals and developing interventions to alter or improve behaviors [10].

### Secondary Email Survey

Findings of the quantitative phase and semistructured interviews were shared with each unit’s clinical staff leaders along with an open-ended 8-question survey (Multimedia Appendix 2). The aim of the follow-up survey was to understand leadership perception and insight pertaining to the results of both steps of the study. This survey was answered by 2 leaders representing 4 main hospital units.

### Analysis

An Excel worksheet (Microsoft Corp) was used to provide frequencies, percentages, and average and total compliance rates. MedCalc for Windows Version 15.0 (MedCalc Software) was used for testing the statistical significance of different associations between morning and evening shift compliance, and reporting odds ratios (OR) and 95% confidence intervals. Qualitative analysis software NVivo Version 12 (QSR International) was used to classify responses under each theme and analyze differences in interview responses depending on the aggregate compliance score of the unit that interviewees were associated with.

Verbal informed consent was obtained from the participants of the qualitative survey. Units were informed of the observational part of the study prior to implementation. Although no personal identifiers of respondents and staff were recorded, the data was kept in secured and password-protected computers. The study was designed as a practice-based quality improvement project; hence, institutional review board approval was not required.

## Results

### Hand Hygiene and Low-Level Disinfection Compliance

The characteristics and activities of the sample of staff observed within each unit are displayed in Table 1. We observed 222 staff members (156 during morning shifts and 66 during evening shifts). Of 526 hand hygiene and 33 low-level disinfection opportunities, compliance was observed 410 (78%) and 17 (51%) times, respectively.

**Table 1:** Characteristics and activities of observed sample.

Shift and unit		Sample of staff observed, n (%)	Hand hygiene		Low-level disinfection	
			Observations, n (%)	Opportunities, n (%)	Observations, n (%)	Opportunities, n (%)
**Morning shift**						
	A	18 (11.5)	40 (13.5)	51 (14.3)	0 (0.0)	1 (4.2)
	B	10 (6.4)	29 (9.8)	31 (8.7)	1 (7.1)	1 (4.2)
	C	21 (13.5)	52 (17.5)	69 (19.3)	2 (14.3)	2 (8.3)
	D	10 (6.4)	16 (5.4)	17 (4.8)	1 (7.1)	1 (4.2)
	E	12 (7.7)	21 (7.1)	32 (9.0)	2 (14.3)	6 (25.0)
	F	11 (7.1)	26 (8.8)	29 (8.1)	1 (7.1)	1 (4.2)
	G	16 (10.3)	16 (5.4)	26 (7.3)	1 (7.1)	5 (20.8)
	H	14 (9.0)	18 (6.1)	22 (6.2)	1 (7.1)	1 (4.2)
	I	10 (6.4)	24 (8.1)	27 (7.6)	0 (0.0)	1 (4.2)
	J	11 (7.1)	18 (6.1)	25 (7.0)	3 (21.4)	3 (12.5)
	K	10 (6.4)	8 (2.7)	11 (3.1)	—^a^	—
	L	13 (8.3)	37 (12.5)	42 (11.8)	2 (14.3)	2 (8.3)
	Morning shift total	156 (100.0)	297 (100.0)	357 (100.0)	14 (100.0)	24 (100.0)
**Night shift**						
	A	11 (16.7)	14 (13.3)	20 (13.8)	0 (0.0)	1 (11.1)
	B	6 (9.1)	11 (10.5)	15 (10.3)	—	—
	C	8 (12.1)	11 (10.5)	15 (10.3)	1 (33.3)	1 (11.1)
	D	7 (10.6)	13 (12.4)	16 (11.0)	2 (66.7)	2 (22.2)
	E	8 (12.1)	16 (15.2)	21 (14.5)	—	—
	F	9 (13.6)	15 (14.3)	18 (12.4)	0 (0.0)	4 (44.4)
	G	7 (10.6)	12 (11.4)	19 (13.1)	—	—
	H	10 (15.2)	13 (12.4)	21 (14.5)	0 (0.0)	1 (11.1)
	Night shift total	66 (100.0)	105 (100.0)	145 (100.0)	3 (100.0)	9 (100.0)
	Grand total	222	402	502	17	33

^a^Not available.

Table 2 and Multimedia Appendix 3 (detailed data) show the compliance scores by each unit. During the day shift, 6 of the 12 observed units had hand hygiene compliance scores less than 0.80 while 5 of the 8 units observed during the night shift had below 0.80 compliance. Low-level disinfection compliance was below 0.80 in 5 of 11 units observed during the day shift and 3 of 5 units observed during the night shift.

We compared the odds of noncompliant behavior in hand hygiene between morning and night shifts by considering the night shift as adverse exposure and assuming that compliance may be lower during evening hours. The odds of noncompliant behavior in hand hygiene were higher among 6 of the 8 observed units; however, this relationship was not statistically significant (Table 3).

**Table 2:** Aggregate unit compliance scores for hand hygiene and low-level disinfection of equipment guidelines.

Unit	Day shift		Night shift		
	Hand hygiene	Low-level disinfection of equipment		Hand hygiene	Low-level disinfection of equipment
A	0.78	0.00		0.70	0.00
B	0.94	1.00		0.73	—^a^
C	0.75	1.00		0.73	1.00
D	0.66	0.29		0.76	—
E	0.94	1.00		0.81	1.00
F	0.90	1.00		0.83	0.00
G	0.62	0.20		0.63	—
H	0.82	0.50		0.65	0.00
I	0.89	0.00		—	—
J	0.72	1.00		—	—
K	0.73	—		—	—
L	0.88	1.00		—	—
Total	0.80	0.54		0.73	0.33

^a^Not available.

**Table 3:** Odd ratios of hand hygiene compliance by night versus morning shift and by observed units.

Unit	Morning shift compliance		Night shift compliance^a^		Odds ratio	95% CI	*P* value
	Yes	No	Yes	No			
A	40	11	14	6	1.56	0.48-5.0	.45
B	29	2	11	4	5.27	0.84-32.9	.07
C	52	17	11	4	1.11	0.31-3.95	.86
D	21	11	16	5	0.59	0.17-2.06	.41
E	16	1	13	3	3.69	0.34-39.83	.28
F	26	3	15	3	1.73	0.31-9.69	.53
G	16	10	12	7	0.93	0.27-3.16	.91
H	18	4	13	7	2.42	0.58-10.03	.22
All units	218	59	105	39	1.37	0.86-2.18	.18

^a^Night shifts were considered adverse exposure and we assumed compliance would be lower during night hours.

### Hand Sanitizer Dispenser Placement (Usability) Factors

The observer determined the usability of hand sanitizer dispensers based on their placement in each unit. The scores were marked down as “0” if the usability factor was not met and “1” if it was adequate. In most units, the dispensers met criteria 2, 5, 6, 7, and 8 but did not meet criteria 1, 3, and 4 (Table 4). All units, except one, had similar usability characteristics; hence, their impact on hand hygiene compliance was not analyzed.

**Table 4:** Hand sanitizer dispenser placement (usability) factors and compliance by units.

Observations and factors		Unit										
		H	G	F	E	D	B	C	A	I	J	K
**Hand sanitizer dispenser placement factors**												
	1. Easily visible on entry	0	0	0	0	0	0	0	0	0	1	0
	2. Easy, unobstructed access	1	1	1	1	1	1	1	1	1	1	1
	3. Close to the point of care	1	1	1	1	1	1	1	0	1	0	1
	4. Visible from point of care	0	0	0	0	0	0	0	0	0	0	0
	5. Along the workflow path	0	0	0	0	0	0	0	1	0	1	0
	6. Close to the entrance or exit	1	1	1	1	1	1	1	1	1	1	1
	7. Placed at optimal height	1	1	1	1	1	1	1	1	1	0	1
	8. Visible on exit	1	1	1	1	1	1	1	1	1	0	1
	Scores	0.625	0.625	0.625	0.625	0.625	0.625	0.625	0.625	0.625	0.5	0.625
**Hand hygiene compliance**												
	Compliance during day shift	0.82	0.62	0.9	0.66	0.94	0.94	0.75	0.78	0.89	0.72	0.73
	Compliance during night shift	0.65	0.63	0.83	0.76	0.81	0.73	0.81	0.7	—^a^	—	—
	Overall compliance	0.735	0.625	0.865	0.71	0.875	0.835	0.78	0.74	0.89	0.72	0.73

^a^Not available.

### Interview Data Findings

In total, 4 staff members, 2 each from units with morning shift compliance ≥0.80 (units B and E) and ≤0.80 (units D and G) consented to interviews. Using the Theoretical Domains Framework to analyze the results from the interview data, we identified several themes across units and compliance levels. Staff had the knowledge and skills to maintain hand hygiene and low-level disinfection of equipment; however, time shortage while seeing patients can make implementation of these skills difficult:

Whenever you’re [a clinical staff member is] trying to get from one place to another it’s easy to...think that you did it.

hand hygiene behavior

It’s sometimes automatic to where you don’t think that you’re doing it and sometimes you can completely skip it.

When we’re shorthanded, yes.

it is difficult to maintain low-level disinfection of equipment

Despite the difficulty, staff members do think of hand hygiene and low-level disinfection as a standard part of their patient consultations. This indicates that they have the self-standard to adhere to the guidelines. Furthermore, staff had the self-efficacy to complete the steps required to perform these behaviors:

It [hand hygiene] just becomes second nature.

It’s [low-level disinfection of equipment is] not difficult at all.

Self-efficacy can become an issue if the patient load is high or time is an issue:

It [hand hygiene] comes down to the time.

It’s [whether or not low-level disinfection behavior is performed is] purely based on staffing, how staffing is and how challenging the patient load.

The staff has greater self-efficacy when they see visual cues such as soiled hands. They were familiar with the consequences of not performing the behavior, with one participant stating that *“Infection rates would go up and I... would get sick more often.”* They also thought that these behaviors are automatic and do not require reminders; however, one respondent said, *“you’re* [a clinical staff member is] *going to forget* [to perform low-level disinfection of equipment] *sometimes but the key is to try to be consistent.”* When specifically asked if these behaviors take up too much time, they generally stated, *“No because those wipes* [which are used for low-level disinfection of equipment] *dry out in like 2 minutes.”* However, *“if we’re* [unit staff members are] *busy and they can’t get to it* [cleaning equipment using low-level disinfection] *then that’s a chore that’s time-consuming.”* This suggests that staff members mostly agree that the environment is conducive to performing these behaviors but that, in a given context, it can take up too much time. Other answers around environmental constraints varied and included the following:

If we’re [unit staff members are] super busy.

It’s [whether or not low-level disinfection of equipment is performed is] going to be staffing issues.

I just think about all the germs and I don’t want to carry that home or give it to anybody else.

Being highly aware of the risks involved with failing to carry out the protocols was the most common answer as to why the work environment motivated staff members to perform a behavior. 

Answers regarding social influences or norms also varied. Some staff members thought that social influences had a role in their hand hygiene behavior, including the following:

Keeping each other accountable.

If you [a clinical staff member] see other people doing it [low-level disinfection of equipment], it helps remind you to do it.

Other staff members indicated that social influences do not correlate with their hand hygiene or low-level disinfection of equipment behavior:

I know it’s [performing low-level disinfection of equipment] automatic…

I do it as necessary so no.

staff do not feel pressured to perform behavior when witnessing other unit members performing it

I know that’s [hand hygiene behavior is] something we have to do and something we should do just because it’s important to keep your hands clean.

In addition, themes surrounding behavioral regulation varied greatly. Staff thought that maintenance of behavior was dependent upon access to supplies:

Just to constantly have the equipment.

needed to perform low-level disinfection

Making sure environmental services keeps the hand sanitizer machines full.

It also depends on time:

Maybe I could wait the 2 minutes I’m supposed to after I clean it.

equipment requiring low-level disinfection

In contrast to behavioral regulation, social norms, and the environmental context domains explored, staff members agreed that emotion is not a factor in hand hygiene or low-level disinfection of equipment behavior. They also agreed that these behaviors are habitual within the given context.

Commenting on whether or not hand sanitizer dispensers located in the hallway, rather than the patient room, would change their hand hygiene habits, a unit G staff member answered, *“I don’t think the hand sanitizer is good to tell you the truth.”* A unit D staff member responded that *“having it outside the room I think you’d* [a clinical staff member would] *see it more.”* These units both had an aggregate compliance score of ≤0.80 for both hand hygiene and low-level disinfection behavior. The unit E interviewee responded with the following:

I feel like that really helps especially if you like sat down and you’re like I forgot to do it, so it’s [the hallway hand sanitizer dispenser is] right there you can always do that.

However, the unit B respondent did not think that any change would occur, stating *“I think it would be the same.”* Units E and B both had an aggregate compliance score of ≥0.80 for both behaviors.

When asked to comment on the changes that could improve low-level disinfection compliance rates, the 2 units with compliance ≤0.80 stated that access and supplies can help:

Only thing I can think of would be…[to]...put it [cleaning supplies needed for low-level disinfection] at every door on the outside of rooms...that way you [a clinical staff member] could literally walk out of the room, grab them, wipe it down, throw them away, go to the next.

Have more purple top wipes [ammonium and alcohol-based wipes] available and bleach wipes.

Suggestions from the other units with compliance ≥0.80 included education and staffing:

They [cleaning staff] literally just don’t understand that they’re supposed to do it [clean certain equipment] so I think like re-education or education initially is a big deal.

Actually have a person here 24/7 to help with cleaning our big equipment.

Regarding the availability of the cleaning supplies for low-level disinfection, staff generally believed that access to them is easy; however, there could be more supplies:

There is easy access to it but there’s no such thing as having too much there’s always room for more.

supplies for low-level disinfection of equipment

There needs to be more because they [hospital supply managers] only put a certain amount on our floor.

Table 5 shows the differences in interview responses given by staff from low- and high-compliance units. Each factor has an aggregate compliance score from 4 units. The scores under each factor were compiled by sorting interviewee responses into 36 relevant categories.

**Table 5:** Compiled responses to interview questions analyzed using NVivo 12^a^.

Factors	Compliance ≥0.80	Compliance ≤0.80
1. Awareness of guidelines	8	8
2. Awareness of risk prompts behavior	8	6
3. Behavior causes drying of hands	2	2
4. Behavior creates burdens	3	5
5. Behavior does not create burdens	5	3
6. Behavior is automatic	15	19
7. Behavior is difficult	0	4
8. Behavior is difficult in the work setting	11	11
9. Behavior is easy	19	17
10. Behavior is easy in the work setting	13	15
11. Behavior is influenced by team members	9	9
12. Behavior is not influenced by team members	8	8
13. Behavior is not time consuming	7	9
14. Behavior is prompted by visual cues	7	3
15. Behavior is standard in work setting	8	8
16. Behavior is time consuming	11	9
17. Behavior requires reminders	13	5
18. Benefits of behavior outweigh burdens	7	5
19. Equipment for behavior is adequate	4	4
20. Equipment for behavior is easily accessible	5	5
21. Equipment for behavior is inadequate	0	2
22. Equipment for behavior is not easily accessible	7	7
23. Guidelines are credible and valid	8	10
24. Improvement (access and supplies)	5	5
25. Improvement (education)	1	3
26. Improvement (increase behavior)	3	3
27. Improvement (staffing)	2	0
28. Improvement (time)	3	1
29. Improvement (visual cues)	0	4
30. Intention to practice behavior	8	8
31. Mood is not a factor	8	4
32. Other team members would agree	5	7
33. Trained in skill of behavior	8	8
34. Understands consequences	8	10
35. Understands guidelines	13	11
36. Understands reasoning behind guidelines	7	9

^a^Results from compliance with low-level disinfection of equipment and hand hygiene behavior were compiled into “behavior” before conducting the analysis.

### Secondary Qualitative Data Results

One theme that emerged from this data was that there might be issues with resources or access to supplies needed to perform these behaviors. For example, one respondent said that “resources are not readily available” and that having holders for the wipe containers in patient rooms could be a viable solution to improve rates of low-level disinfection behavior. In addition, the respondent believed another way of improving compliance scores would be to hold team members accountable. When asked about processes that are in place to maintain these behaviors, the respondents referred to the survey readiness audits, suggesting that these are effective in monitoring behavior. Another process that was considered effective is the Stop the Line program, which urges staff members to speak up in situations where they feel that patient safety is at risk. Having more hand sanitizer dispensers in hallways as opposed to primarily in patient rooms was also proposed as a solution to improve hand hygiene compliance.

## Discussion

In this study, we found that 6 (50%) and 2 (25%) units had over 80% (0.80) hand hygiene compliance during the morning and evening shifts, respectively. Aggregated low-level disinfection compliance was 0.54 during the morning shift and 0.33 during the evening shift. The odds of noncompliant hand hygiene behavior were 1.4 times higher among staff who worked night shifts compared to those who worked day shifts; however, this relationship was not statistically significant (*P*=.45). All units, except one, had similar hand sanitizer dispenser usability characteristics. During the qualitative part of the study, some identified challenges included the following: “shortage of time while seeing patients,” “some time staff forgets” [sic], “concern about drying hands,” “behavior is difficult or require reminders” [sic], and “there may be issues with resources or access to supplies to perform these behaviors.” Staff also stated that “a process that is considered effective is the Stop the Line program,” and that the “behavior is easy and automatic.”

Observation results reflected compliance levels that are comparable to the findings from other studies [2,3,11]. An increase in hand hygiene compliance could reduce nosocomial infection rates and lead to improved patient safety [12,13]. Although qualitative data does provide some insight into the cognitive aspects of behavior, further investigation as to why compliance scores are less than optimal in certain areas is needed. The observer noted that higher compliance scores were connected with several behaviors or traits of the unit, including the ability or willingness to accept criticism or feedback from peers. In addition, we found that high compliance scores were associated with task ownership and overall accountability as a team. There is a well-documented association between a good team environment and the promotion of health care worker behavior that is associated with reduced risk to patient safety [14-16]. Furthermore, adherence and commitment to organizational processes can also improve staff compliance positively [17].

Interview responses associated with different levels of compliance as analyzed using NVivo 12 also indicated what kind of intervention components need to be implemented. Clinical staff members from units with compliance scores over 0.80 tended to be more willing to admit that the behaviors were not automatic (Table 5, item 6) and reminders were needed to maintain them (Table 5, item 17). They indicated actively using visual cues as reminders (Table 5, item 14), which is in contrast to clinical staff members from units with compliance scores below 0.80, who stated that visual cues could be used to improve behavior (Table 4, item 29). Additionally, clinical staff members from units with compliance scores over 0.80 did not indicate that the behavior was ever difficult, whereas clinical staff members from units with scores below 0.80 indicated that the behavior was difficult at times. The combination of these differences suggests that reminders are needed to ensure higher levels of compliance.

In addition, qualitative methods revealed data that is consistent with other studies that assessed health care workers’ attitudes toward these behaviors [18]. When asked about barriers to hand hygiene behavior, two unit members commented that the products can cause drying of hands; in addition, time constraints within the work setting were consistently mentioned by respondents. This is consistent with findings by Kirk et al [18].

Limitations of our study include difficulties with observations (eg, not being able to witness all observations due to the room doors being closed, a long walking distance from one patient to the next, and observing without intruding on medical practice. Semistructured interviews and email survey samples were relatively small; thus, the points of view shared by respondents may not be generalizable to the whole facility or another institution. However, these two methods still revealed important information.

In summary, hand hygiene and low-level disinfection of equipment compliance is dependent on several personal and nonpersonal factors. Issues such as time constraints, peer pressure, work culture, available resources, and understanding of guidelines were found to be most connected with staff behavior and consistent with existing literature [19]. Novel approaches such as sanitizer-dispensing door handles can improve hand hygiene practices and compliance [20]. Furthermore, the working culture and environment in a health care setting can influence staff behavior as well as patient safety outcomes [21].

## Appendix

Multimedia Appendix 1 Interview guide.

Multimedia Appendix 2 Qualitative data collected via email.

Multimedia Appendix 3 Results from the observation of hand hygiene and low-level disinfection of equipment.

## Abbreviations

CDI: *Clostridioides difficile* infections
HAI: hospital-acquired infections
SIR: Standard Infection Ratio
