# Supplementary material for: Assessing Hand Hygiene and Low-Level Disinfection of Equipment Compliance in an Acute Care Setting: Mixed Methods Approach
Source: JMIR Nurs. 2020 Jun 5;3(1):e18788. doi: 10.2196/18788 (PMC8279436; doi:10.2196/18788)
Supplement: Multimedia Appendix 1 [file nursing_v3i1e18788_app1.docx]

**APPENDIX 1**

**Interview Guide**

***Hand Hygiene***

Do you understand and consent that this interview is completely anonymous and only the unit number and your answers will be recorded? We only ask that you be honest to the best of your knowledge.

**Unit _____ Participant Consent _____**

1. Are you aware of any guidelines about hand hygiene?
2. If yes, what do those guidelines include?
3. If yes, why are these guidelines in place?
4. Were you ever trained in the proper technique for hand hygiene?
5. Is hand hygiene easy or difficult to maintain when you are seeing patients?
6. Is hand hygiene a standard part of your patient consultations?
7. What do you think about the credibility of the guidelines?
8. Do you think that other people in your unit would agree?
9. How easy or difficult is it for you to practice hand hygiene?
10. What makes it easy or difficult?
11. What would happen if you did not practice hand hygiene?
12. What burdens does maintaining good hand hygiene create?
13. Do the benefits outweigh these burdens?
14. Do you intend to practice good hand hygiene?
15. Is hand hygiene something you do automatically or do you need to be reminded?
16. Do you ever feel that practicing good hand hygiene takes up too much time?
17. Do other team members influence your decision to practice good hand hygiene?
18. What aspects of your work environment influence whether you practice good hand hygiene?
19. When other people in your unit practice hand hygiene do you feel pressured to do so as well?
20. Does your mood affect whether or not you practice hand hygiene?
21. Are there procedures or ways of working that encourage good hand hygiene?
22. What could you personally do better to practice good hand hygiene?
23. Are there any rooms that you can think of in the unit that have the hand sanitizer placement in a different location to the other rooms?
24. If there were more hand sanitizer dispensers in the hall, do you think this would change your hand hygiene habits?

***Low-level Disinfection of Equipment***

Do you understand and consent that this interview is completely anonymous and only the unit number and your answers will be recorded? We only ask that you be honest to the best of your knowledge.

**Unit _____ Participant Consent _____**

1. Are you aware of any guidelines about low level disinfection of equipment?
2. If yes, what do those guidelines include?
3. If yes, why are these guidelines in place?
4. Were you ever trained in the proper technique for low level disinfection of equipment?
5. Is low level disinfection of equipment easy or difficult to maintain when you are seeing patients?
6. Is low level disinfection of equipment a standard part of your patient consultations?
7. What do you think about the credibility of the guidelines?
8. Do you think that other people in your unit would agree?
9. How easy or difficult is it for you to practice low level disinfection of equipment?
10. What makes it easy or difficult?
11. What would happen if you did not practice low level disinfection of equipment?
12. What burdens does maintaining low level disinfection of equipment create?
13. Do the benefits outweigh these burdens?
14. Do you intend to practice low level disinfection of equipment?
15. Is low level disinfection of equipment something you do automatically, or do you need to be reminded?
16. Do you ever feel that practicing low level disinfection of equipment takes up too much time?
17. Do other team members influence your decision to practice low level disinfection of equipment?
18. What aspects of your work environment influence whether you practice low level disinfection of equipment?
19. When other people in your unit practice low level disinfection of equipment do you feel pressured to do so as well?
20. Does your mood affect whether or not you practice low level disinfection of equipment?
21. Are there procedures or ways of working that encourage low level disinfection of equipment?
22. What could you personally do better to practice low level disinfection of equipment?
23. What do you think we could change to make it easier to practice low level disinfection of equipment?
24. Do you find that there is often easy access to the purple wipes or does there need to be more available?
