# Supplementary material for: Assessing Hand Hygiene and Low-Level Disinfection of Equipment Compliance in an Acute Care Setting: Mixed Methods Approach
Source: JMIR Nurs. 2020 Jun 5;3(1):e18788. doi: 10.2196/18788 (PMC8279436; doi:10.2196/18788)
Supplement: Multimedia Appendix 2 [file nursing_v3i1e18788_app2.docx]

**APPENDIX 2**

**Qualitative Data Collected via Email**

1. Do the results from your unit surprise you?
2. Why/why not?
3. What do you think contributes to your unit's compliance score?
4. What are your initial thoughts on ways to maintain or improve your unit's compliance score?
5. What are the challenges that you or your unit have faced in maintaining adherence to the guidelines provided?
6. Approximately how often do you think you have to remind team members to perform these behaviors? (once a month, once a week, several times a week, once a day, several times a day)
7. As a unit, do you have processes in place to uphold hand hygiene and low-level disinfection of equipment guidelines?
8. If yes, what are they? If no, do you think implementing a process would change your compliance score?
