# Supplementary material for: Assessing Hand Hygiene and Low-Level Disinfection of Equipment Compliance in an Acute Care Setting: Mixed Methods Approach
Source: JMIR Nurs. 2020 Jun 5;3(1):e18788. doi: 10.2196/18788 (PMC8279436; doi:10.2196/18788)
Supplement: Multimedia Appendix 3 [file nursing_v3i1e18788_app3.docx]

**APPENDIX 3**

**Observation of Hand Hygiene and Low-Level Disinfection of Equipment Results**

| **Hand Hygiene and Low-Level Disinfection Data** | **A** | **B** | **C** | **D** | **E** | **F** | **G** | **H** | **I** | **J** | **K** | **L** | **TOTAL** |
| --- | --- | --- | --- | --- | --- | --- | --- | --- | --- | --- | --- | --- | --- |
| **# of Hand Sanitizer Dispensers in Hall** | 5 | 4 | 5 | 6 | 5 | 6 | 6 | 6 | 18 | 3 | 12 | N/A | 76 |
| **Day Shift Time Observed** | 9:00 AM | 11:25 AM | 10:00 AM | 10:20 AM | 11:15 AM | 10:10 AM | 11:15 AM | 10:10 AM | 11:20 AM | 11:50 AM | 10:50 AM | 11:10 AM |  |
| **Aggregate Day Shift Score Hand Hygiene** | 0.78 | 0.94 | 0.75 | 0.66 | 0.94 | 0.9 | 0.62 | 0.82 | 0.89 | 0.72 | 0.73 | 0.88 | 0.8 |
| **# of Compliant Behaviors Observed** | 40 | 29 | 52 | 21 | 16 | 26 | 16 | 18 | 24 | 18 | 8 | 37 | 305 |
| **# of Opportunities for Compliance Observed** | 51 | 31 | 69 | 32 | 17 | 29 | 26 | 22 | 27 | 25 | 11 | 42 | 382 |
| **Aggregate Day Shift Score Low-Level Disinfection** | 0 | 1 | 1 | 0.29 | 1 | 1 | 0.2 | 0.5 | 0 | 1 | N/A | 1 | 0.54 |
| **# of Compliant Behaviors Observed** | 0 | 1 | 2 | 2 | 1 | 1 | 1 | 1 | 0 | 3 | NO | 2 | 14 |
| **# of Opportunities for Compliance Observed** | 1 | 1 | 2 | 7 | 1 | 1 | 5 | 2 | 1 | 3 | NO | 2 | 26 |
| **Night Shift Time Observed** | 9:40 PM | 10:10 PM | 9:30 PM | 7:20 PM | 10:50 PM | 7:40 PM | 8:20 PM | 7:00 PM |  |  |  |  |  |
| **Aggregate Night Shift Score Hand Hygiene** | 0.7 | 0.73 | 0.73 | 0.76 | 0.81 | 0.83 | 0.63 | 0.65 | N/A | N/A | N/A | N/A | 0.73 |
| **# of Compliant Behaviors Observed** | 14 | 11 | 11 | 16 | 13 | 15 | 12 | 13 | NO | NO | NO | NO | 105 |
| **# of Opportunities for Compliance Observed** | 20 | 15 | 15 | 21 | 16 | 18 | 19 | 20 | NO | NO | NO | NO | 144 |
| **Aggregate Night Shift Score Low-Level Disinfection** | 0 | N/A | 1 | N/A | 1 | 0 | N/A | 0 | N/A | N/A | N/A | N/A | 0.33 |
| **# of Compliant Behaviors Observed** | 0 | NO | 1 | NO | 2 | 0 | NO | 0 | NO | NO | NO | NO | 3 |
| **# of Opportunities for Compliance Observed** | 1 | NO | 1 | NO | 2 | 4 | NO | 1 | NO | NO | NO | NO | 9 |
